# Heat absorption effect of magneto-natural convection flow in vertical concentric annuli with influence of radial and induced magnetic field

**DOI:** 10.1038/s41598-024-64779-x

**Published:** 2024-07-02

**Authors:** Muhammad Yusuf Muhammad, Muhammad Auwal Lawan, Yusuf Y. Gambo

**Affiliations:** 1 Department of Mathematics, Aliko Dangote University of Science and Technolgy, Wudil, Kano, 713101 Nigeria; 2 Department of Mathematics, Yusuf Maitama Sule University, Kano, 700103 Nigeria

**Keywords:** Magneto-natural convection, Heat absorption, Radial and induced magnetic field, MHD, Mathematics and computing, Applied mathematics

## Abstract

This paper aims to study the natural convective magneto-hydrodynamic flow of fluid through vertical concentric annuli with iso-flux heating under the conditions of constant internal heat absorption and an induced magnetic field. By solving the set of dimensionless coupled governing equations, we were able to obtain exact expressions for the temperature field, velocity field, and induced magnetic field. We also managed to derive the formulas for skin friction, mass flux, and induced current density. We also examined the effects of non-dimensional parameters on skin friction and mass flux. For easy comprehension and interpretation, the results are provided graphically and in tabular form. The heat absorption parameter, the induced current density, the induced magnetic field, and velocity exhibit a negative trend as the Hartmann number (*Ha*) value increases. The induced magnetic field has the effect of raising both the induced current density and velocity profile. It is found that, when a fluid absorbs heat, the heat absorption parameter experiences reverse flow. For the heat-absorbing fluids, the radii ratio has the effect of increasing velocity, induced magnetic field, and induced current density. The numerical values of skin friction and mass flux at cylindrical walls increase (decrease) with increasing heat absorption parameter and generally it has decreasing tendency with increasing Hartmann number

## Introduction

Heat exchanger analysis and design frequently make use of the annular geometry. Researchers are paying more attention to the study of transport phenomena in the context of annular geometry because of its many applications in both geophysics and engineering, including the design of magnetohydrodynamic (MHD) power generators, the study of geothermal sources, the treatment of nuclear fuel debris, and the optimization of processes for the solidification of metals and metal alloys. There are numerous applications for studying natural convective flow along a vertical cylinder in fields like technology, agriculture, oceanography, and geothermal power generation. Applications in solar power collectors, magnetohydrodynamic power generator design, and oil thermal recovery have drawn attention to the study of transport phenomena with annular geometry. One possible application of magnetohydrodynamics is to pinch the heated plasma. In oceanography, an MHD flow meter, whose induced voltage is proportionate to the flow rate, measures ship speed. By implementing a heat source/sink in a concentric annulus, we can maximize fluid flow.^1^. Couette^2^ conducted the first study on the flow of viscous incompressible fluid in concentric annuli to determine the fluid viscosity.

Studying MHD-natural convection interactions in open-ended vertical concentric annuli helps in optimizing the cooling systems of nuclear reactors to enhance safety and efficiency^3^. Analyzing the influence of radial and induced magnetic fields on fluid flow and heat transfer in vertical annuli aids in the development of more efficient geothermal energy extraction techniques^4^. Research on open-ended vertical concentric annuli provides insights into the complex fluid dynamics involved in MHD propulsion applications^5^. Investigating the influence of radial and induced magnetic fields in vertical annuli helps in optimizing these industrial processes for improved productivity and energy efficiency^6^. By studying the interaction between natural convection and magnetic fields in vertical annuli, researchers can develop advanced MHD systems for harnessing energy from natural sources more effectively^7^.^8^ examined MHD natural convection in a horizontal annulus, highlighting the role of magnetic field strength on flow stability and heat transfer enhancement.^9^ conducted numerical simulations to analyze MHD natural convection in a square enclosure, emphasizing the importance of magnetic Prandtl number in determining flow regimes and heat transfer characteristics.

A new method was developed by^10–13^ analyzed natural convection flows using a new method to derive the approximate equations. The natural convection flow of magneto-hydrodynamic fluid has drawn interest from a wide range of researchers due to its numerous applications in geophysics, astrophysics, meteorology, aerodynamics, magneto-hydrodynamic power generators and pumps, boundary layer control energy generators, accelerators, aerodynamic heating, polymer technology, petroleum industry, crude oil purification, and material processing operations like extrusion, metal forming, continuous casting wire, and glass fiber drawing. By using this method, the well-known Boussinesq approximation was confirmed in the case of a given Newtonian liquid or gas, also known as the constant property model. Four different reference temperatures were used to introduce Boussinesq approximations for the model and properties. However, power law correlations and the state equations of an ideal gas were used to calculate the density and the transport properties.

Magnetohydrodynamics, or MHD, is the fusion of classical electromagnetism and fluid mechanics. In the magneto-hydrodynamics domain, fluids can be propelled and controlled by the Lorentz body forces that arise from the interaction of electric currents and magnetic fields in the presence of an external magnetic field. Motion perpendicular to a magnetic field imposed on an electrically conducting fluid is retarded due to its significant effect^14,15^.

Several physical problems of practical interest, such as convection in the Earth’s mantle, post-accident heat removal, fire and combustion modeling, fluids undergoing exothermic/endothermic chemical reactions, development of metal waste from spent nuclear fuel, applications in the field of nuclear energy, and so forth, have heat transfer features that are significantly influenced by properties related to heat absorption and generation.^16^ investigated the influence of heat-generating/absorbing fluid on heat transfer and transitively on flow to understand the flow of this type of fluid. This is because the volumetric heat generation/absorption term may have significant effects on heat transfer as the temperature rises.

By ignoring the induced magnetic field,^10,17–20^ has compared the magnetohydrodynamics velocity with the classical velocity between two rotating co-axial cylinders in the presence of a radial magnetic field.

Many discussions have focused on the less productive area of ignoring the induced magnetic field’s effect. For example, the velocity functional profile without the induced magnetic field’s effect is smaller than that with one; this suggests that the induced magnetic field increased velocity and induced current density, as some literature has explained^20–24^.

A great deal of physical phenomena involves natural convection that is fueled by heat absorption. This phenomenon is important for several physical issues, including those involving chemical reactions and fluid dissociation. Heat absorption effects can change the temperature distribution, which lowers the rate of particle deposition. Applications about nuclear reactor cores, electronic chips, semiconductor wafers, fire and combustion modeling, and so on may experience this^8^. Identified that the radial magnetic field significantly alters the flow patterns and heat transfer characteristics within the concentric cylinder configuration. The presence of the magnetic field affects the stability of the flow and leads to changes in temperature distribution^25^, conducted numerical simulations to investigate the behavior of MHD natural convection flow with radial magnetic fields. Their findings revealed that the magnetic field induces additional fluid motion, resulting in enhanced heat transfer rates compared to cases without magnetic fields.^26^ Analyzed heat transfer enhancement mechanisms in MHD natural convection flow with radial magnetic fields. They observed that the presence of the magnetic field alters the boundary layer thickness and enhances convective heat transfer near the inner and outer cylinders.

The impact of an induced magnetic field on fully formed convection flow in an annular micro-channel was examined by^27^. It is found that there is more skin friction at the annular micro-channel surface in the presence of an induced magnetic field.^28^ have performed two-dimensional laminar flow of an incompressible fluid across a surface. They discovered that the local Nusselt number depends on the wall suction velocity and Hartmann number.^29^ used the finite volume method to examine the laminar convection flow of an electrically conducting fluid while taking heat absorption and generation into account. The average Nusselt number is shown to decrease when internal heat generation is present. Heat generation and absorption’s effects on Casson fluid flow across a vertical.

Examining fully developed magneto-natural convectional flow in vertical annuli while taking constant iso-flux heating into account was done by^30–32^. This is because the cylinder’s boundary surface is constantly heated, which causes a lot of thermal energy differential and modifies the model’s system. Detailed analysis and observation of the unified influence for iso-flux conditions for the temperature field at the inlet cylinder is to be done by^17^

The current work illustrates the problem of heat absorption’s effects on natural convection flow along a vertical coaxial cylinder with constant iso-flux heating, which has not been previously investigated. The heating type at the inner cylinder’s inlet in the current heat absorption process is the main focus of this investigation. Analytical solutions are obtained for dimensionless ordinary differential equations, which are a convenient form of basic ordinary differential equations. The findings enable us to forecast the distinct behavior that becomes visible upon modification of the pertinent parameters.

### Mathematical formulation

The basic hydrodynamic equations and Maxwell’s electromagnetic equations for electrically charged fluid flow in steady state^1,33–36^. These equations in vector form are as follows:

Continuity equation:

1 $$\begin{aligned} \nabla \cdot V =0 \end{aligned}$$

Momentum equation:

2 $$\begin{aligned} \rho (\nabla \cdot V)V =-\nabla P+\mu (\nabla ^{2}V)+(J\times B)+\rho g \end{aligned}$$

Magnetic field equation:

3 $$\begin{aligned} (\nabla ^{2}H)+\nabla \times (J\times B)=0 \end{aligned}$$

Energy equation:

4 $$\begin{aligned} (V\cdot \nabla )T=\frac{k}{\rho C_{p}}\nabla ^{2}T+\frac{Q_{o}}{\rho C_{p}} \end{aligned}$$

**Figure 1:**
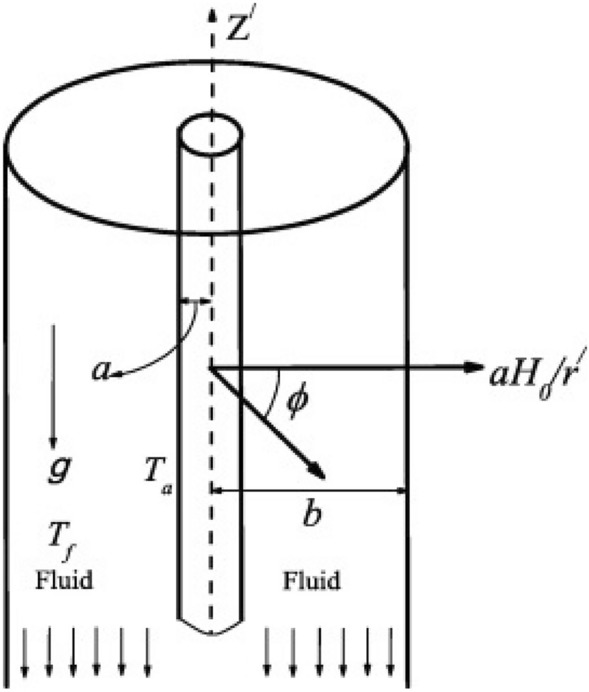
Geometry of the model.

The configuration of the study is sketched in Fig. 1 We have considered a fully developed natural convective flow of a steady, laminar, viscous, and incompressible electrically conducting fluid between vertical concentric annulus of infinite length. the $z^{'}$ -axis is taken along the axis of the co-axial cylinder and measured in the vertically upward direction, and $r^{'}$ is the radial direction measured outward from the axis of the cylinder. $aH_{o}^{'}/r^{'}$ is the applied magnetic field directed radially outward, $T_{w}^{'}$ and $T_{f}^{'}$ denote the temperature at the outer surface of the inner cylinder and the ambient temperature respectively. Also, *a* and *b* are taken as the radius of the inner and outer cylinders respectively. $U_{r^{'}}^{'}, U_{\theta ^{'}}^{'}$ and $U_{z^{'}}^{'}$ are the velocity components in $r^{'}, \theta ^{'}$, and $z^{'}$ directions respectively. Also, $U_{r^{'}}^{'}= U_{\theta ^{'}}^{'}=0$ since the flow is fully developed and $z^{'}$ is the direction of the flow along the axis of concentric cylinders. The variables describing the flow formation depend only on the coordinate $r^{'}$ because the flow is fully developed and the cylinders are of infinite length. for the considered model, the components of velocity and magnetic field are respectively given as $\left( 0, 0, U_{r^{'}}^{'}(r^{'})\right)$ and $\left( aH_{o}^{'}/r^{'}, 0, H_{z^{'}}^{'}(r^{'})\right)$.

As indicated in the Fig. 1, the component of *V* and *H* are $\left( 0, 0, U_{r^{'}}^{'}(r^{'})\right)$ and $\left( aH_{o}^{'}/r^{'}, 0, H_{z^{'}}^{'}(r^{'})\right)$ respectively

The gradient and Laplacian operators are given by Eqs. (5) and (6)

5 $$\begin{aligned} \nabla = \frac{\partial }{\partial r}\textbf{e}_r + \frac{1}{r}\frac{\partial }{\partial \theta }\textbf{e}_\theta + \frac{\partial }{\partial z}\textbf{e}_z \end{aligned}$$

6 $$\begin{aligned} \nabla ^{2}=\frac{1}{r}\frac{\partial }{\partial r}\left( r\frac{\partial }{\partial r}\right) +\frac{1}{r^{2}}\frac{\partial ^{2}}{\partial \theta ^{2}}+\frac{\partial ^{2}}{\partial z^{2}} \end{aligned}$$

Utilizing $\nabla ^{2}$ and $\nabla$ to the velocity *U*(*r*) and magnetic field *H*(*r*) and to the $\nabla \times \left( V \times H\right)$ and $J\times B$ respectively we get Eqs. (7)–(10)

7 $$\begin{aligned} \nabla ^{2} V=\left[ \dfrac{1}{r^{'}}\dfrac{d}{dr^{'}}\left( r^{'}\dfrac{dU^{'}(r^{'})}{dr^{'}}\right) \right] \end{aligned}$$

8 $$\begin{aligned} \nabla ^{2} H=\left[ \dfrac{1}{r^{'}}\dfrac{d}{dr^{'}}\left( r^{'}\dfrac{dH_{z^{'}}^{'}(r^{'})}{dr^{'}}\right) \right] \end{aligned}$$

9 $$\begin{aligned} \nabla \times \left( V \times H\right) = \begin{vmatrix} \textbf{r}&\mathbf {\theta }&\textbf{z} \\ \frac{\partial }{\partial r}&0&0 \\ 0&0&-\frac{aH_oU(r)}{r} \end{vmatrix}= \frac{aH_o}{r}\frac{dU(r)}{dr} =\dfrac{aH_{o}^{'}}{r^{'}}\left( \dfrac{dU(r)^{'}}{dr^{'}}\right) \end{aligned}$$

Similarly,

10 $$\begin{aligned} J\times B= \mu _{e} \left( \nabla \times H\right) \times H= \dfrac{a\mu _{e}H_{o}^{'}}{r^{'}}\dfrac{dH_{z^{'}}^{'}}{dr^{'}} \end{aligned}$$

The basic governing equations for the flow formation utilizing Eqs. (7)–(10) in Eqs. (1)–(4) and invoking the Boussinesq approximation the equations are as follows:

11 $$\begin{aligned} \nu \left[ \dfrac{1}{r^{'}}\dfrac{d}{dr^{'}}\left( r^{'}\dfrac{dU^{'}(r)}{dr^{'}}\right) \right] +g\beta (T^{'}-T^{'}_{f})+\dfrac{a\mu _{e}H_{0}^{'}}{\rho r^{'}} \left( \dfrac{d H_{z^{'}}^{'}(r)}{dr^{'}}\right) =0 \end{aligned}$$

12 $$\begin{aligned} \eta \left[ \dfrac{1}{r^{'}}\dfrac{d}{dr^{'}}\left( r^{'}\dfrac{dH_{z^{'}}^{'}(r)}{dr^{'}}\right) \right] +\dfrac{a H_{0}^{'}}{r^{'}}\left( \dfrac{d U^{'}(r)}{dr^{'}}\right) =0 \end{aligned}$$

13 $$\begin{aligned} \dfrac{\kappa }{\rho C_{p}}\left[ \dfrac{1}{r^{'}}\dfrac{d}{dr^{'}}\left( r^{'}\dfrac{dT^{'}(r)}{dr^{'}}\right) \right] +\dfrac{Q_{0}(T^{'}-T^{'}_{f})}{\rho C_{p}}=0 \end{aligned}$$

The boundary conditions for the Eqs. 11), (12) and (13) are given as follows:

14 $$\begin{aligned} \begin{aligned} {\left\{ \begin{array}{ll} U^{'}(r)=H_{z^{'}}^{'}(r)=0 \quad \dfrac{d\, T^{'}(r)}{d\,r^{'}}=C \quad \text {at}\quad r^{'}=a \\ \quad \\ U^{'}(r)=H_{z^{'}}^{'}(r)=0 \quad T^{'}(r)=T_{f}^{'} \quad \text {at}\quad r^{'}= b \end{array}\right. } \end{aligned} \end{aligned}$$

In the above equations, the fluid velocity, acceleration due to gravity, coefficient of volume expansion, magnetic permeability, density of the fluid, magnetic diffusivity, thermal conductivity of the fluid, specific heat at constant pressure, temperature of the fluid, ambient temperature, and the heat absorption for $Q_{o} < 0$ are represented by the following: $U^{'}, g, \beta , \mu _{e}, \rho , \eta , C_{p}, T^{'}, T_{f}^{'}$, and $Q_{o}$ respectively.

By non-dimensionalizing Eqs. (11), (12), (13) and (14) using the following non-dimensional variables:

$$u=U^{'}/U$$,    $$r=r^{'}/a$$ ,   $$\lambda =b/a$$,    $$T=(T^{'}-T_{f}^{'})/(T_{w}^{'}-T_{f}^{'})$$    $$H=(H_{z^{'}}^{'})/(\sigma a\mu _{e}H_{o}^{'}U)$$

also, utilizing some non-dimensional parameters such as Hatmann number, Characteristic velocity of the fluid and heat generation/absorption parameter the governing equations of the flow in dimensionless is as follows:

$$Ha=\mu _{e}H_{o}^{'}\sqrt{\frac{\sigma }{\rho \nu }}$$    $$U=g\beta a^{2}(T_{w}^{'}-T_{f}^{'})/\nu$$    $$S=Q_{o}/k(T_{w}^{'}-T_{f}^{'})$$ we get

15 $$\begin{aligned} \dfrac{1}{r}\dfrac{d}{dr}\left( r\dfrac{dU(r)}{dr} \right) + T(r)+\dfrac{Ha^{2}}{r}\left( \dfrac{d H(r)}{dr}\right) =0 \end{aligned}$$

16 $$\begin{aligned} \dfrac{1}{r}\dfrac{d}{dr}\left( r\dfrac{dH(r)}{dr} \right) +\dfrac{1}{r}\left( \dfrac{d U(r)}{dr}\right) =0 \end{aligned}$$

17 $$\begin{aligned} \dfrac{1}{r}\dfrac{d}{dr}\left( r\dfrac{dT(r)}{dr} \right) -S=0 \end{aligned}$$

Subject to the boundary conditions:

18 $$\begin{aligned} \begin{aligned} {\left\{ \begin{array}{ll} U(r)=H(r)=0; \quad \dfrac{d\, T(r)}{d\,r}=-1 \quad \text {at}\quad r=1 \\ \quad \\ U(r)=H(r)=0; \quad T(r)=0 \quad \text {at}\quad r= \lambda \end{array}\right. } \end{aligned} \end{aligned}$$

## Methods

The methodology adopted in this study is similar to one presented in^1,32^. The analytical solution for the velocity, induced magnetic field and temperature field is obtained by solving the non-dimensional governing linear simultaneous ordinary differential eqautions utilizing non-dimensional boundary condition and numerical values were also obtained.

### Analytical solution

Solving Eqs. (15)–(17) analytically subject to boundary conditions (18), the velocity, induced magnetic field and temperature field were obtained respectively as follows:

Solving energy equation

19 $$\begin{aligned} U(r)=C_{1}r^{Ha}+C_{2}r^{-Ha}+C_{4}+(A_{5}+A_{2}ln(r)r^{2})+A_{4}Sr^{4} \end{aligned}$$

20 $$\begin{aligned} H(r)=C_{3}-(C_{1}r^{Ha}-C_{2}r^{-Ha})/Ha+(A_{6}+A_{7}ln(r))r^{2}+A_{8}Sr^{4} \end{aligned}$$

21 $$\begin{aligned} T(r)=C_{5}ln(r)+C_{6}-Sr^{2}/4 \end{aligned}$$

The formulae of skin friction, induced current density, induced current flux and mass flux are given in Eqs. (20), (21), (22) and (23) respectively

The skin-frictions at the outer surface of the inner cylinder and the inner surface of the outer cylinder in non-dimensional form are obtained:

22 $$\begin{aligned} \tau _{1}=\left. \dfrac{dU}{dr} \right| _{1}=Ha(C_{1}-C_{2})+A_{60}\qquad \text {and}\qquad \tau _{\lambda }=\left. \dfrac{dU}{dr} \right| _{\lambda }=A_{61}C_{1}+C_{2}A_{62}+A_{61} \end{aligned}$$

Using Maxwell’s equation, the induced current density along the $\theta$-direction can be found using Eq. (20) as follows:

23 $$\begin{aligned} \begin{aligned} J_{\theta }(r)=&-\left( \dfrac{dH(r)}{dr}\right) \\ =&(C_{1}r^{Ha-1}+C_{2}r^{-Ha-1})+(A_{5}+A_{2}ln(r))r+A_{4}Sr^{3} \end{aligned} \end{aligned}$$

Eqs. (19) and (20) are used to determine the mass flux and the induced current flux of the fluid through the annuli, respectively:

24 $$\begin{aligned} Q=2\pi \int ^{\lambda }_{1}U(r)\,dr =2\pi (A_{73}+C_{3}A_{43}) \end{aligned}$$

25 $$\begin{aligned} J=\int ^{\lambda }_{1}J_{\theta }(r)\,dr=A_{47}+C_{3}A_{45} \end{aligned}$$

The constants in Eqs. (19)–(25) $A_i \text { for } i=1,2,3,4,\ldots ,90$ and $C_i \text { for } i=1,2,3,4,5,6$ are stated in as:

$$\begin{aligned} \begin{array}{lrlr} A_{1}=4C_{5}/(4-Ha^{2}) &{} A_{2}=-C_{5}/(4-Ha^{2}) &{} A_{3}=-C_{6}/(4-Ha^{2}) &{} A_{4}=1/(16-Ha^{2}) \\ A_{5}=A_{1}+A_{3} &{} A_{6}=(A_{2}/4-A_{5}/2) &{}A_{7}=A_{2}/2&{} A_{8}=-A_{4}/4 \\ A_{9}=(\lambda ^{Ha}-1) &{} A_{10}=(\lambda ^{-Ha}-1) &{} A_{11}=A_{5}(\lambda ^{2}-1) &{}A_{12}=A_{2}\lambda log(\lambda )\\ A_{13}=A_{4}S(\lambda ^{4}-1) &{} A_{14}=A_{11}+A_{12}+A_{13} &{} A_{15}=-(A_{5}+A_{4}S) &{} A_{16}=A_{6}+A_{8}S \\ A_{17}=-(E_{1}+E_{2}/Ha) &{} A_{18}=(E_{2/Ha-E_{1}}) &{} A_{19}=E_{1}A_{15}+E_{2}A_{16} &{} A_{20}=-E_{5}A_{5}\lambda \\ A_{21}=-E_{5}A_{2}\lambda log(\lambda ) &{} A_{22}=E_{5}A_{4}S\lambda ^{3} &{} A_{23}=A_{20}+A_{21}+A_{22} &{} A_{24}=E_{6}\lambda ^{2}A_{6}log(\lambda ) \\ A_{26}=E_{6}A_{8}S\lambda ^{4} &{} A_{27}=A_{24}+A_{25}+A_{26} &{} A_{28}=-(E_{5}\lambda ^{M-1}+E_{6}\lambda ^{Ha}/Ha) &{} A_{29}=-(E_{5}\lambda ^{-1-Ha}/Ha) \\ A_{30}=A_{23}+A_{27} &{} A_{31}=(E_{2}A_{28}-E_{6}A_{17}) &{} A_{32}=(E_{2}A_{29}-E_{6}A_{18}) &{} A_{33}=(E_{2}A_{30}-E_{6}A_{19}) \\ A_{34}=(A_{9}A_{32}-A_{10}A_{31}-A_{10}A_{31}) &{} A_{35}=(A_{9}A_{35}-A_{14}A_{31}) &{} A_{36}=-A_{35}/A_{34} &{} A_{37}=(A_{31}A_{10}-A_{9}A_{32}) \\ A_{39}=-A_{38}/A_{39} &{} A_{40}=-S(E_{3}+E_{4}/2)/2 &{} A_{41}=log(\lambda ) &{} A_{42}=-S\lambda ^{2}/4 \\ A_{43}=(E_{4}A_{41}-E_{3}) &{} A_{44}=(A_{40}A_{41}-E_{3}A_{42}) &{} A_{45}=CA_{41} &{} A_{46}=A_{45}-A_{44} \\ A_{47}=A_{46}/A_{43} &{} A_{48}=-(A_{47}+A_{42}) &{} A_{49}=A_{48}/A_{41} &{} A_{64}=A_{58}\lambda log(\lambda )\\ A_{51}=1-A_{42} &{} A_{58}=2A_{2} &{} A_{59}=4A_{4} &{} A_{54}=A_{53}/A_{52} \\ A_{55}=A_{54}-A_{50} &{} A_{57}=-2A_{5}+A_{2} &{} A_{60}=A_{57}+A_{59}S &{} A_{61}=N \lambda ^{Ha-1} \\ A_{62}=-N \lambda ^{-N-1} &{} A_{63}=A_{57}\lambda &{} A_{68}=A_{58}\lambda log(\lambda ) &{} A_{66}=A_{63}+A_{64}+A_{65} \\ A_{67}=(\lambda ^{Ha+2}-1)/Ha+2 &{} A_{68}=(\lambda ^{2-HA}-1)/(2-hA) &{} A_{69}=(\lambda ^{2}-1)/2 &{} A_{70}=A_{5}(\lambda ^{4}-1)/4 \\ A_{71}=A_{2}\lambda ^{4}log(\lambda )/4 &{} A_{72}=-A_{2}(A_{2}(\lambda ^{4})/16) &{} A_{73}=A_{4}S(\lambda ^{6}-1)/6 &{} A_{74}=A_{70}+A_{71}\\ A_{75}=A_{72}+A_{73} &{} A_{76}=C_{1}A_{67}+C_{2}A_{68} &{} A_{77}=C_{4}A_{69}+A_{79}+A_{75} &{} A_{78}=A_{76}+A_{77} \\ A_{85}=(\lambda ^{Ha}-1)/Ha &{} A_{80}=-(\lambda ^{-Ha}-1)/Ha &{} A_{82}=A_{4}S(\lambda ^{4}-1)/4 &{} A_{83}=A_{2}\lambda ^{2}log(\lambda )/2 \\ A_{84}=-A_{2}(\lambda ^{2}-1)/4 &{} A_{85}=A_{81}+A_{83} &{} A_{86}=A_{84}+A_{82} &{} A_{87}=A_{85}+A_{86} \\ A_{88}=C_{1}A_{79}+c_{2}A_{80} &{} A_{89}=A_{88}+A_{87} &{} C_{1}=A_{39} &{} C_{2}=A_{36} \\ C_{3}=-(C_{1}A_{28}+C_{2}A_{29}+A_{30})/E_{6} &{} C_{5}=A_{49} &{} C_{6}=A_{47} &{} C_{6}=A_{47} \\ C_{4}=-(C_{1}+C_{2}+A_{90}) &{} A_{90}=A_{5}+A_{4}S &{}&{} \end{array} \end{aligned}$$

## Results and discussion

**Figure 2:**
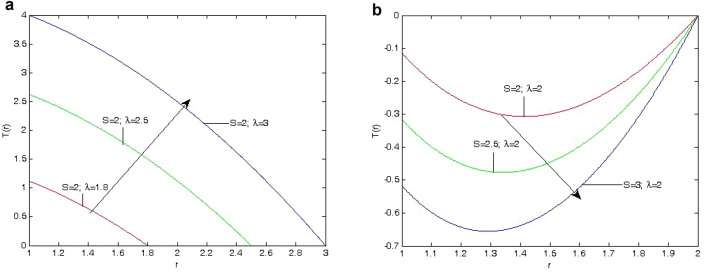
(**a**) Effect of the annular gap $\lambda =1.8,2.5\; \& \; 3$ on the temperature field *T*(*r*) at $S=2.0$. (**b**) Effect of the heat absorption parameter $(S=2.0, 2.5 \; \& \; 3)$ on the temperature field *T*(*r*) at $\lambda =2$.

**Figure 3:**
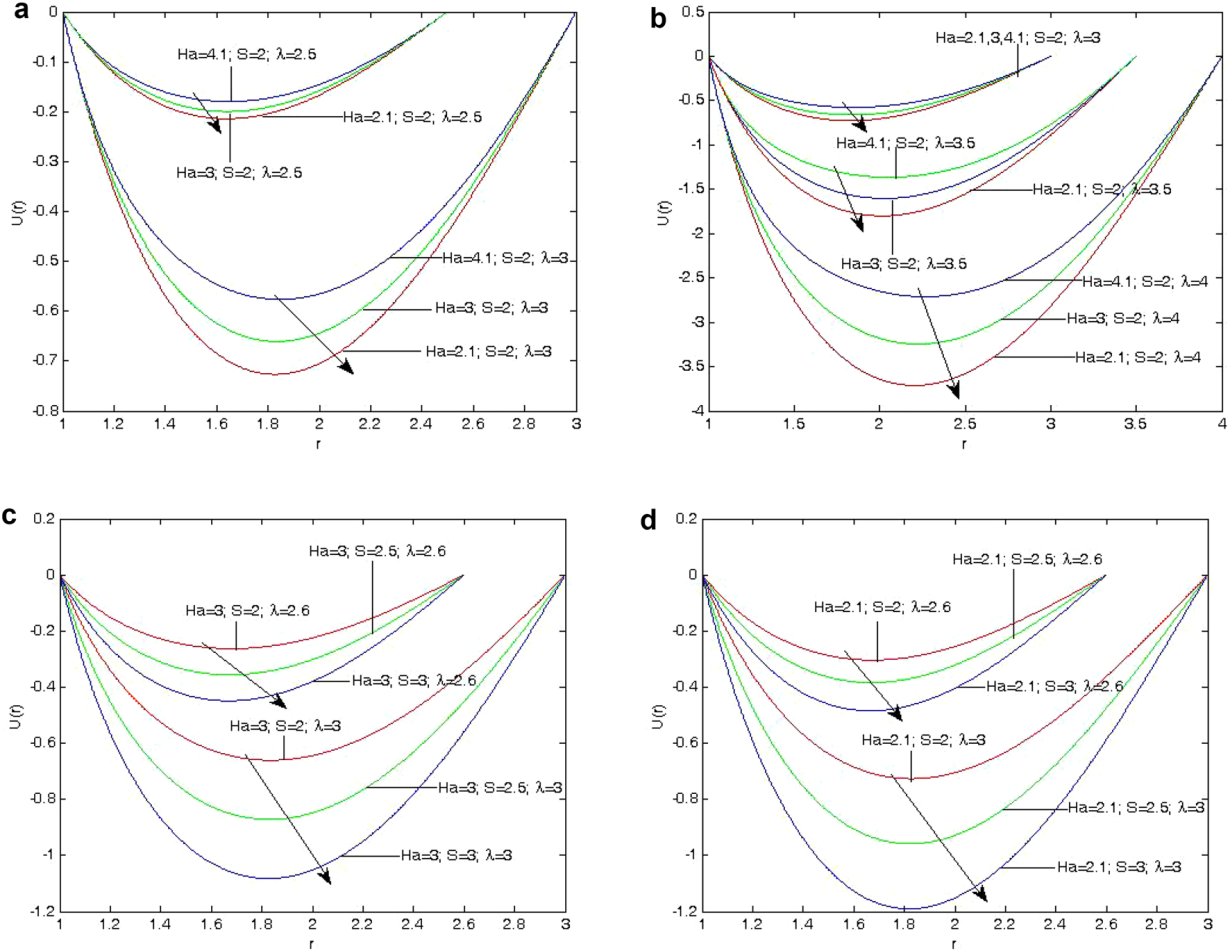
(**a**) Variation of velocity profile *U*(*r*) on different values of *Ha* with $\lambda =2.5, 3$ at $S=2.0$. (**b**) Variation of velocity profile *U*(*r*) on different values of *Ha* and $\lambda$ at $S=2.0$. (**c**) Variation of velocity profile *U*(*r*) on different values of *S* with $\lambda =2.6, 3$ at $Ha=3$. d. Variation of velocity profile *U*(*r*) on different values of *S* with $\lambda =2.6, 3$ at $Ha=2.1$.

**Figure 4:**
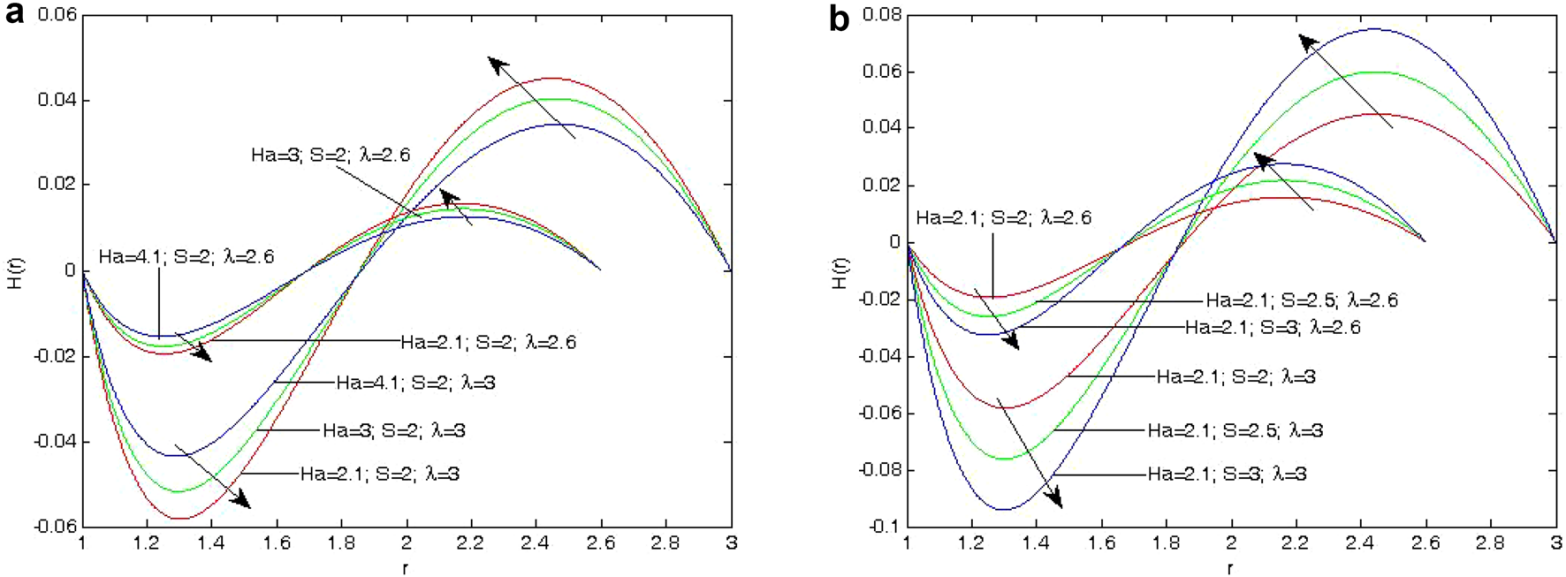
(**a**) Variation of Induced magnetic field *H*(*r*) on different values of *Ha* and $\lambda$ at $S=2$. (**b**) Variation of Induced magnetic field *H*(*r*) on different values of *Ha* and *S* with $\lambda = 2.6,3$.

**Figure 5:**
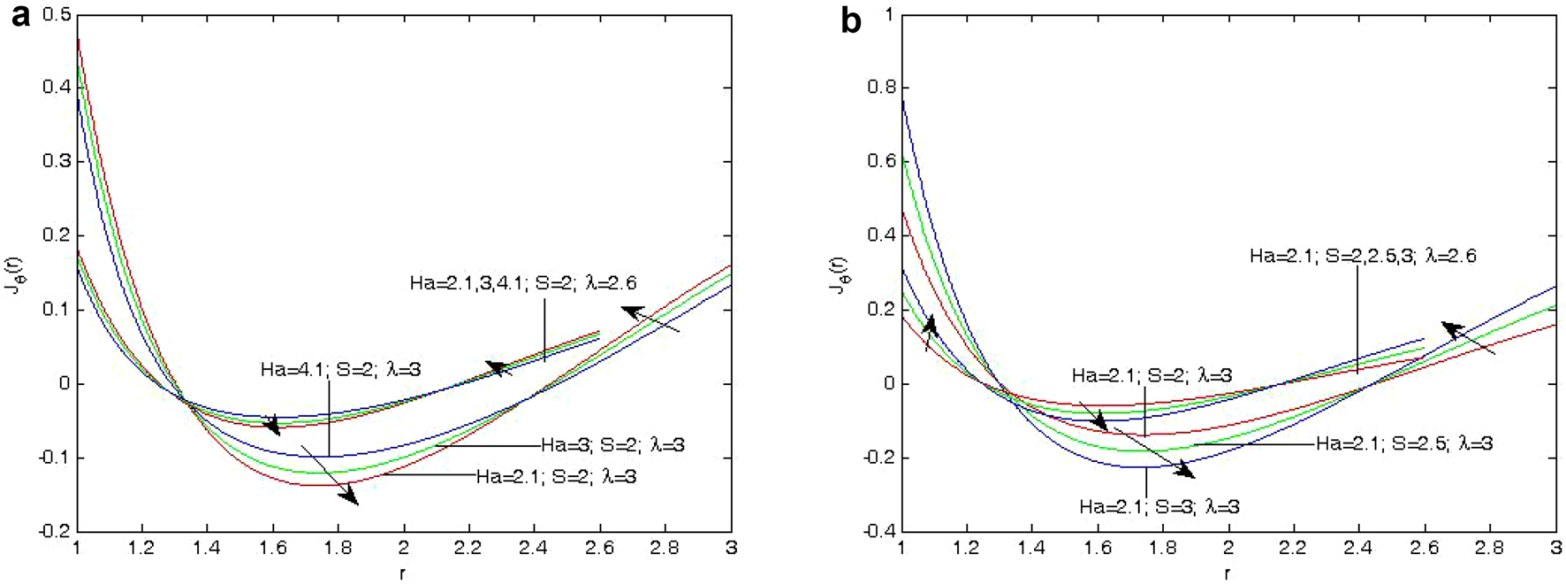
(**a**) Variation of Induced current density $J_{\theta }$ on different values of *Ha* and $\lambda$ at $S=2.0$ (**b**) Variation of Induced current density $J_{\theta }$ on different values of *Ha* and *S* at $\lambda$.

To assess the fluid flow, the governing equations were analytically solved, and the results were plotted using MATLAB. The analysis of the findings was predicated on the three parameters-heat absorption, radii ratio, and Hartmann number which were employed in this work. After analyzing each parameter while keeping the others constant, the graphs were generated as in Figs. 2a, 3, 4 and 5b. The numerical computations of Eqs. (20)–(23) are given in the Table 1 and the figures are illustrated below:

The effects of the fluid flow parameters on velocity, induced magnetic field, temperature, skin friction, mass flux, and induced current density have been shown using graphs as shown in Figs. 2a, 3, 4 and 5b. These results show the variations in the velocity, induced magnetic field, temperature, skin friction, mass flux, and induced current density, as influenced by the parameters of the fliud flow problem, that is, Hartmann number (*Ha*) , heat absorption parameter (*S*), and annular gap.

The present work conduct a study analysis by considering the influence of radial and induced magnetic field with iso-flux at the inner cylinder on a steady fully developed magneto-natural convection flow of viscous, incompressible, electrically conducting fluid in a vertical concentric annuli. The values of Hartmann number (*Ha*) are taken over the range of $2\le Ha\le 5$ as in^1^.

The expression for the velocity, induced magnetic field and temperature profile in Eqs. (19), (20) and (21). The effect of annular gap $(\lambda )$, Heat absorption parameter (*S*) and Hartmann number (*Ha*) are similar as those given by^1^.

Fig. 2a, b illustrate the effects on the temperature field of the heat absorption parameter (*S*) and the ratio of the outer radius to the inner radius $(\lambda )$. The temperature field for heat absorption is enhanced by the outer radius to inner radius ratio. Additionally, it has been shown that decreasing the thickness of the thermal boundary layer causes an increase in temperature profiles when the heat absorption parameter rises.

In the heat absorption, Fig. 3a, b indicate that fluid velocity falls as the heat absorption parameter (*S*) increases as this causes a reduction in the thickness of the thermal boundary layer or a decrease in internal heat energy. Furthermore, as the Hartmann number *Ha* increases, it is evident from Fig. 3a, b that the velocity decreases since the Lorentz force increases with increasing the Hartmann number (*Ha*).

The velocity profile decreases due to the influence of the magnetic field when Lorentz force is present, as it reduces the force on the velocity field. This indicates the velocity is decreased by the Hartmann number *Ha*. Studying Fig. 3c shows the ratio of the outer to inner radius $(\lambda )$ influences the fluid’s velocity because it causes the annular space between the two cylinders to rise.

In this section, we have observed the influence of the Hartmann number, the ratio of outer radius to inner radius, and the heat absorption parameter on the induced magnetic field. Figure 4a shows that the induced magnetic field increases and decreases as values of the heat absorption parameter (*S*) increase for heat absorption due to enhancing the thickness of the thermal boundary layer reducing as the heat absorption parameter (*S*) increases.

The increase in the value of the Hartmann number (*Ha*) implies that the induced magnetic field decreases since the Lorentz force acts in the opposite direction of the induced magnetic field. The ratio of outer radius to inner radius $(\lambda )$ is to increase the induced magnetic field profiles, since the gap between two cylinders increases, in heat absorption In Fig. 5a, b, we have analyzed the effect of heat absorption parameter (*S*) on the induced current density $J_\theta$. It indicates that the induced current density $J_\theta$ decreases with an increase in heat absorption parameter (*S*). It is also clear from Fig. 5a, b that the induced current density $J_\theta$. has a decreasing tendency with increasing value of the Hartmann number (*Ha*) due to the presence of Lorentz force. This also indicates that the induced current density profiles have an increasing nature with increasing value of the ratio of outer radius to inner radius, due to increment in the annulus space between both the cylinders.

The numerical values of the skin-friction at the inner and outer surfaces of the cylinders, as well as the mass flow and induced current flux of the fluid, are represented by Table 2. As the heat absorption parameter rises, the skin-friction and mass flux also rise with a rising ratio of outer radius to inner radius. When the Hartmann number at the inner and outer cylinders increases for heat absorption for any value of annular gab, the skin-friction reduces. As the annular gap grows positively, the mass flux tends to increase as the Hartmann number and heat absorption parameter increase. When the ratio of outer to inner radius, heat absorption parameter, and Hartmann number increase, the induced current exhibits an oscillating behavior. Table 1 shows the comparison between the current study and the work of Kumar and Singh^1^.

The influence of the parameters in the flow formation provides valuable insights into the physical significance of these factors. Here are some implications

- Influence of Hartmann number: higher Hartmann numbers decrease velocity, induced magnetic field, and induced current density, indicating suppression of convective flow and magnetic effects.
- Effect of induced magnetic field and heat generation/absorption: induced magnetic field enhances velocity and induced current density, while heat generation increases these parameters and heat absorption leads to reverse flow, illustrating the interplay between magnetic fields and thermal effects.
- Impact of heat absorption parameter and cylinder geometry: increasing heat absorption parameter decreases velocity, induced magnetic field, induced current density, and temperature, whereas increasing outer radius to inner radius ratio enhances these parameters, underscoring the significance of thermal effects and geometric configurations.
- Skin friction and mass flux: skin friction tends to increase (decrease) with increasing heat absorption parameter and generally decreases with increasing Hartmann number, indicating the influence of thermal and magnetic conditions on surface interactions and fluid transport properties.

**Table 1:** Comparison of skin-frictions by neglecting iso-flux heating effects.

Ha	$S=2.0,\lambda =1.8$ $\tau _0$		$S=2.0,\lambda =1.8$ $\tau _1$	
	Reference^1^	Present results	Reference^1^	Present results
2.1	0.320407	0.32041	0.140259	0.14026
3.0	0.319661	0.31966	0.140673	0.14067
4.1	0.318704	0.31870	0.141205	0.14121

Ha	$S=2.0,\lambda =1.8$ $\tau _0$		$S=2.0,\lambda =1.8$ $\tau _1$	
	Reference^1^	Present results	Reference^1^	Present results
2.1	0.442544	0.44255	0.193723	0.19373
3.0	0.442087	0.44208	0.195951	0.19596
4.1	0.441508	0.44151	0.196241	0.19624

**Table 2:** Numerical values for the skin friction, mass flux, and induced current flux in dimensionless form.

$\lambda$	*S*	*Ha*	$\tau _{1}$	$\tau _{\lambda }$
1.8	2.0	2.1	0.3526	$-0.0594$
		3.0	0.3518	$-0.0333$
		4.1	0.3506	$-0.0170$
2.0		2.1	0.6116	$-0.0678$
		3.0	0.6105	$-0.0356$
		4.1	0.6087	$-0.0165$
3.0		2.1	3.8253	$-0.1064$
		3.0	3.8527	$-0.0418$
		4.1	3.8875	$-0.0131$
1.8	3.0	2.1	0.4499	$-0.0733$
		3.0	0.4489	$-0.0415$
		4.1	0.4475	$-0.0213$
2.0		2.1	0.8002	$-0.0867$
		3.0	0.7990	$-0.0458$
		4.1	0.7972	$-0.0214$
3.0		2.1	5.3533	$-0.1482$
		3.0	5.3953	$-0.0583$
		4.1	5.4485	$-0.0184$

$\lambda$	*S*	*Ha*	*Q*	*J*
1.8	2.0	2.1	0.2218	$8.8818\times 10^{-16}$
		3.0	0.2163	$1.1102\times 10^{-16}$
		4.1	0.2078	$2.2204\times 10^{-16}$
2.0		2.1	0.6244	$8.8818\times 10^{-16}$
		3.0	0.6035	$-2.2204\times 10^{-16}$
		4.1	0.5720	0.0000
3.0		2.1	17.8222	$7.1054\times 10^{-15}$
		3.0	16.5342	0.0000
		4.1	14.8540	0.0000
1.8	3.0	2.1	0.2870	$3.5527\times 10^{-15}$
		3.0	0.2798	$1.1102\times 10^{-16}$
		4.1	0.2688	$2.2204\times 10^{-16}$
2.0		2.1	0.8280	$-1.0658\times 10^{-14}$
		3.0	0.8002	$-2.2204\times 10^{-16}$
		4.1	0.7584	$-4.4409\times 10^{-16}$
3.0		2.1	25.1702	0.0000
		3.0	23.3500	$-8.8818\times 10^{-16}$
		4.1	20.9755	0.0000

## Conclusion

The study of steady fully developed magneto-natural convection flow in vertical concentric coaxial cylinders under the influence of a radial and induced magnetic field and heat absorption have been investigated. Based on the provided figures and numerical data, it is possible to draw the simple conclusion as follows:

- The velocity, induced magnetic field and induced current density have decreasing tendency with increase in the value of Hartmann number in the absorption
- The induced current density and velocity profile both rise as a result of the induced magnetic field. When a fluid is producing heat, the heat generation and absorption parameter increases the velocity, induced magnetic field, and induced current density; when a fluid is absorbing heat, the reverse flow occurs.
- The effect of induced magnetic field is to increase the velocity, magnetic field and induced current density profiles.
- The effect of heat absorption parameter is to decreases the values of velocity, induced magnetic field, induced current density and temperature.
- The effect of ratio of outer radius to inner radius is to increase the velocity, induced magnetic field, induced current density and temperature field in the heat absorption.
- Also, the numerical values of skin friction and mass flux at cylindrical walls increase (decrease) with increasing heat absorption parameter and generally it has decreasing tendency with increasing Hartmann number Overall, the conclusions drawn from the study provide valuable insights into the complex interplay between magnetic fields, thermal effects, and geometric configurations on fluid flow behavior in vertical concentric annuli, offering potential avenues for further research and practical applications in various engineering and scientific fields.

## Data Availability

The dataset for all analysis involved in this manuscript is available from the corresponding author on reasonable request.

## List of symbols

*a*: Radius of the inner cylinder (m)
*b*: Radius of the outer surface (m)
*g*: Acceleration due to gravity ( m s$^{-2}$)
$$H^{'}_{0}$$: Induced magnetic field (kg A$$^{-1}$$ s$$^{-2})$$
$$H_{z^{'}}^{'}$$: Induced magnetic field (kg A$$^{-1}$$ s$$^{-2})$$
*H*: Dimensionless induced magnetic field
*h*: Convective heat transfer coefficient (W m$$^{-2}$$ K$$^{-1})$$
$$C_{p}$$: Specific heat capacity at constant pressure (J kg$$^{-1}$$ K$$^{-1})$$
$$J_{\theta }$$: Induced current density (Am$$^{-2})$$
*Ha*: Hartmann number
$$q^{''}$$: Heat flux (W $$m^{-2})$$
$r^{'}$: Cylindrical ordinate in r-direction (m)
$z^{'}$: Cylindrical ordinate in z-direction (m)
*r*: Dimensionless radial distance
$T^{,}$: Temperature of the fluid (K)
*T*: Dimensionless temperature of the fluid
$$T_{f}^{'}$$: Ambient temperature
$T_{w}^{'}$: Temperature of the outer surface of the inner cylinder (K)
$$U^{'}$$: Velocity along the axial direction (m s$$^{-1})$$
*U*: Dimensionless fluid velocity
$U_{0}$: Characteristic velocity along the axial velocity m s$^{-1}$
$$Q_{0}$$: volumetric rate of the heat generation (or absorption) (W m$$^{-3})$$
*S*: Dimensionless heat generation (or absorption)

## Greek symbol

$$\kappa$$: Thermal conductivity of the fluid (m s$$^{-2})$$
$$\eta$$: Magnetic diffusivity (m s$$^{-2})$$
$$\beta$$: Coefficient of thermal expansion (K$$^{-1})$$
$$\nu$$: Kinematic viscosity of the fluid (kg m$$^{-1}$$ s$$^{-1})$$
$$\mu _{e}$$: Magnetic permeability (kg A$$^{2}$$ m s$$^{-2})$$
$$\rho$$: Density of the fluids (kg m$$^{-3})$$
$$\lambda$$: Annular gap
$\sigma$: Conductivity of the fluid $(\Omega ^{-1} \textrm{m}^{-1}\; \textrm{or}\; \textrm{A}^{2} \, \textrm{kg}^{-1}\, \textrm{s}^{3}\, \textrm{m}^{-3})$
$\tau _{\lambda }$: Skin friction coefficient outer radius
$\tau _{1}$: Skin friction coefficient inner radius
